# The clinical prognostic significance of hs-cTnT elevation in patients with acute ischemic stroke

**DOI:** 10.1186/s12883-018-1121-5

**Published:** 2018-08-20

**Authors:** Lanying He, Jian Wang, Weiwei Dong

**Affiliations:** 1 Department of Neurology, The Second People’s Hospital of Chengdu, Chengdu, 400010 People’s Republic of China; 2 Department of Neurology, First Affiliated Hospital, Chongqing Medical University, Chongqing, China, 400030 People’s Republic of China

**Keywords:** Acute ischemic stroke, Heart damage, Hs-cTnT,electrocardiographic abnormality, Insular stroke

## Abstract

**Background:**

Cardiac autonomic dysfunction caused by ischemic stroke might lead to an adverse outcome. Elevated high sensitivity cardiac troponin (hs-cTnT) is a marker of cardiac disease, it can elevate in acute stroke patients. The aim of the present study was to investigate association between serum hs-cTnT with prognosis among patients with acute ischemic stroke.

**Methods:**

Five hundred and sixteen patients (mean age 66.19 ± 10.11) with acute ischemic stroke underwent a comprehensive clinical investigation and serum hs-cTnT activity test. All patients were followed up for 3 months. The prognosis was death or major disability (modified Rankin Scale score ≥ 3) at 3 months after acute ischemic stroke.

**Results:**

22.87% (118/516) of patients had serum hs-cTnT elevation (≥14 ng/l). Compared with normal hs-TnT group, the incidence of insular stroke (adjusted odds ratio, 2.84; 95% confidence interval, 1.48–4.17; *P* = 0.001) were more likely in patients with hs-cTnT elevation. In fully adjusted models, there was an association between serum hs-cTnT elevation and death (adjusted odds ratio, 3.14; 95% confidence interval, 1.16–8.49; *P* = 0.02) and major disability(adjusted odds ratio, 2.07; 95% confidence interval, 1.04–4.51; *P* = 0.04), and composite outcome(adjusted odds ratio,2.22;95% confidence interval,1.10–4.48; *P* = 0.03).

**Conclusions:**

Higher levels of serum hs-cTnT were independently associated with increased risk of death or major disability after stroke onset, suggesting that serum hs-cTnT may have prognostic value in poor outcomes of ischemic stroke.

## Background

Stroke is an important contributor to death and major disability. Ischemic stroke is the most common subtype of stroke. Autonomic dysfunction is a frequent in stroke patients. Studies showed that cardiac autonomic dysfunction caused by ischemic stroke might lead to an adverse outcome. Cardiac comorbidities account for almost 20% of deaths after ischemic stroke, appropriate preventive or therapeutic measures can be taken if the patient with acute stroke at risk of myocardial injury could be identified at the time of admission. High sensitivity cardiac troponin T (hs-cTnT) is the most sensitive marker of myocardial injury, however it can rise in several other conditions (e.g. renal failure, sepsis, heart failure, and pulmonary edema) [1, 2]. In the last decade, the importance of hs-cTnT elevated in acute stroke has attracted many scholars’ interest. Previous studies have shown that serum hs-cTnT is elevated in 10–30% of acute stroke patients [3–5]. In addition, some studies have found that elevated serum hs-cTnT levels may be associated with specific areas of brain damage [6]. However, it still remains unclear the pathomechanism of hs-cTnT elevation during the acute stage of ischemic stroke, which may be developed by neurally mediated autonomic dysregulation after acute stroke. In the other hand, in published studies, there were small numbers of patients. To date, whether hs-cTnT levels is associated with death or poor outcome remain uncertain [7, 8].

To test the hypothesis that if serum hs-cTnT levels can help predict cardiac complications and poor outcome in acute ischaemic stroke(AIS), we studied the prognostic correlates of elevated hs-cTnT levels on admission in a cohort of consecutive patients.

## Methods

### Study population

The consecutive patients who were admitted to the Second People’s Hospital of Chengdu due to AIS within 72 h of symptom onset between May 2012 and December 2017. Stroke patients were diagnosed as AIS if the brain computed tomography (CT) scan was normal or showed acute ischemic changes according to World Health Organization definition (sudden neurological deficit with a putative vascular cause). Acute ischemic stroke was confirmed by diffusion-weighted imaging (DWI) magnetic resonance imaging (MRI) using Siemens Magnetom Avanto 1.5 Tesla (Siemens Medical Solutions, Erlangen, Germany). The severity of stroke was assessed by National Institutes of Health Stroke Scale score(NIHSS). Eligible patients were all patients admitted to our stroke unit during the study period. This study was approved by ethics committee. Written informed consent was obtained from all study participants or their legal proxies.

### Inclusion and exclusion criteria

Patients were included in the study only if they fulfilled all the following criteria:1. Admission for first-ever acute ischemic stroke.2. Evidence of a single acute hemispheric lesion consistent with clinical manifestations. 3. Cardiac(include acute myocardial infarction, congestive heart failure, a history of tachyarrhythmia/bradyarrhythmia or atrial fibrillation),pulmonary disease and impaired renal function (estimated glomerularfiltration rate < 60 mL/min per 1.73 m^2^) were excluded.4. Any pharmacological treatment, including β-blockers, possibly affecting the autonomic function were excluded. 5.Cerebral hemorrhage, fever, hypoxia also were excluded. No patients received mechanical thrombectomy and thrombolytic therapy. All patients received standard therapy, which consisted of aspirin, lipid-lowering medications and so on. All patients were followed up for 3 months. The outcome was defined as death and major disability (scores 3–5 of modified Rankin Scale [mRS]) at 3 months after stroke onset.

### Data collection

CT or MRI examination was conducted at the time of admission, and repeated examination was performed on 5 days after admission to confirm the location of the lesion. The presence of insular infarction was assessed by an experienced neuroradiologist blinded to clinical details.

Serum hs-cTnT was measured as part of routine laboratory testing on admission, which were measured by Elecsys and cobase analyzer (Roche diagnostics). Levels of serum hs-cTnT was considered abnormal if it was≥14 ng/l.

Standard 12-lead electrocardiogram (ECGs) examination was performed on admission and assessed by two inspectors, blinded for patients. The Data differences between observers were resolved by consensus.

After the heart investigation, if the patients were suspected to have acute coronary syndrome, the cardiologist would perform additional cardiac evaluations for the patients. The waveforms of 12-lead ECGs were uploaded in digitai form, and explained by a cardiologist. Two dimensional transthoracic echocardiography was performed on patients with suspected reversible cardiac ischemia, and then we excluded patients with reversible cardiac ischemia.

### Statistical analysis

Firstly, patients were classified into normal and hs-cTnT elevation group according to the level of serum hs-cTnT on admission. Demographic characteristics, vascular risk factors, current smoke, and so on were compared between the 2 subgroups in univariate analysis, using Pearson χ 2 test, Fisher exact 2-sided test, or Student t test, mean values(±standard deviation) were calculated for continuous variables. Mann-Whitney U test was used to test differences between two groups. We then performed logistic regressions analyses to determine the association between serum hs-cTnT and outcome(death, major disability and death/major disability), adjusting for age, sex, hypertension, current Smoking, current alcohol drinking, diabetes, hyperlipidemia, insular stroke, family history of stroke and NIHSS score. Results were expressed as adjusted odds ratios (OR) with the corresponding 95% confidence interval (CI).The data were analyzed using SPSS software (SPASS 22.0). *P* values< 0.05 were considered as statistically significant.

## Results

### Characteristics of the study subjects

During the study period, 516 patients were identified, comprised 49.03%(253) men and 50.97%(263) women, and the mean age was 66.19 ± 10.11 years(38-96 years). In the study population, 367 patients had a history of hypertension, 154 had a history of diabetes, 274 had a history of hyperlipidemia, 149 patients smoke,153patients current alcohol drinking. Of these patients, 152were diagnosed as insular stroke. During the 3-month follow-up period, 49 out of 516(9.49%) patients had died.

### Univariable models for predictors of elevated hs-cTnT

Serum hs-cTnT levels were normal in 398 (77.13%) patients, the elevated hs-cTnT level in 118 (22.87%). Baseline characteristics of patients in the normal hs-cTnT group(< 14 ng/L) and the elevated hs-cTnT group(≥14 ng/l) were compared (Table 1). Patients with elevated hs-cTnT showed significantly higher prevalence of older age, insular stroke and high NIHSS score than patients with normal hs-cTnT (both *P* < 0.05). In multivariate logistic regression, insular stroke associated with hs-cTnT elevation (adjusted odds ratio, 2.48; 95% confidence interval, 1.48–4.17; *P* = 0.00).

**Table 1:** Comparison of baseline characteristics between patients with normal and elevated hs-cTnT group

	hs-cTnT< 14 ng/L (398)	hs-cTnT≥14 ng/L (118)	OR(95%CI)	P*
Age, y(mean,SD)	65.36 ± 10.07	69.01 ± 9.77		*0.00*
Females,n(%)	205 (51.51)	58(49.15)	0.91 (0.60–1.37)	0.65
Men,n(%)	193(48.49)	60(50.85)	0.91(0.60–1.37)	0.65
Hypertension,patients,n(%)	287(72.11)	80(67.80)	0.81(0.52–1.27)	0.36
Current Smoking,n(%)	111(27.89)	38(32.20)	1.23(0.79–1.92)	0.36
Current alcohol drinking,n(%)	115(28.89)	38(32.20)	1.17(0.75–1.82)	0.49
Diabetes, n(%)	115(28.89)	39(33.05)	1.22(0.78–1.89)	0.39
Hyperlipidemia,n(%)	214(53.77)	60(50.85)	0.89(0.59–1.34)	0.58
ST/T change, n(%)	110 (27.64)	39(33.05)	1.293(0.83–2.01)	0.23
Insular stroke,n(%)	89(22.36)	63(53.39)	3.98(2.58–6.12)	*0.00*
Family history of stroke, n(%)	74(18,59)	27(22.88)	1.30(0.79–2.14)	0.30
NIHSS score(min-max)	2–25	2–30		
NIHSS score(mean,SD)	8.82 ± 4.39	11.80 ± 5.21		*0.00*

*Comparison between normal hs-cTnT group and elevated hs-cTnT group

### Multivariable models on the association between elevated hs-cTnT and prognosis

Mortality rates were 24.58%(29/118) in the elevated hs-cTnT group at 3 months, which was significantly higher than that in the normal hs-cTnT group(5.03%,20/398) (*P* = 0.000). In addition to 49 dead, the remaining 467 patients, major disability rates were 40.45%(36/89) in the elevated hs-cTnT group at 3 months, which was significantly higher than that in the normal hs-cTnT group(17.99%,68/378) (*P* = 0.000). After adjusting for age, sex, hypertension, current smoking, current alcohol drinking, diabetes, hyperlipidemia, insular stroke, family history of stroke and NIHSS score on admission,3-month mortality in the elevated hs-cTnT group was higher than in the normal hs-cTnT group (adjusted odds ratio, 3.14; 95% confidence interval, 1.16–8.49; *P* = 0.02), and 3-month major disability in the elevated hs-cTnT group was higher than in the normal hs-cTnT group (odds ratio, 2.17; 95% confidence interval, 1.04–4.51; *P* = 0.04). After adjusting for fully confounders, the combined prognosis of death/disability was significantly higher for the elevated hs-cTnT group at 3 months(adjusted odds ratio, 2.22; 95% confidence interval, 1.10–4.48; *P* = 0.03).(Table 2).

**Table 2:** Multivariable Models Showing Association Between elevated hs-cTnT and Prognosis

	OR (95% CI)	P*
death	3.14(1.16–8.49)	0.02
Major disability(mRS 3–5)	2.17(1.04–4.51)	0.04
Major disability(mRS3–5) + death	2.22(1.10–4.48)	0.03

*Multivariable adjusted for age, sex, hypertension, current Smoking, current alcohol drinking, diabetes, hyperlipidemia, insular stroke, family history of stroke and NIHSS score

In this study, we also found that the concentration of hs-cTnT were significantly correlated with poor prognosis, the higher the hs-cTnT, the worse the prognosis. The levels of hs-cTnT in the death group and the survival group were respectively 18.67 ± 10.39, 10.26 ± 6.85 (*P* = 0.00), the levels of hs-cTnT in the mRS ≤ 2 group and the major disability group were respectively 9.14 ± 5.98, 14.14 ± 8.15(P = 0.00),and the levels of hs-cTnT in the mRS ≤ 2 group and the composite outcome group were respectively 9.14 ± 5.98, 15.59 ± 9.14(P = 0.00).

## Discussion

Hs-cTnT is the most sensitive and specific biomarker of myocardial injury,which is widely used in the diagnosis of the patients with heart diseases, especially in patients with non-ST segment elevation acute coronary syndrome [9, 10]. Many studies have shown that serum Hs-cTnT of many patients with acute stroke increases significantly. The current treatment guidelines for acute ischemic stroke patients recommend troponin evaluation in acute stage [11]. It is still controversial whether the increase of troponin after AIS is related to the mortality and disability rate of stroke patients. Most studies suggest that there is a link between them, but a few studies that hold the opposite view. Some studies have shown that elevated troponin is related to poor functional prognosis, and high troponin levels is associated with increased mortality [12–15]. The potential pathophysiological mechanism of troponin elevation in the AIS is still unclear, leading to considerable uncertainty in the diagnosis and treatment for the clinician. In our study, 22.87%(118/516) of acute stroke patients had elevated serum hs-cTnT level which was congruent with previous studies. Mortality rates and major disability rate in the elevated hs-cTnT group respectively were 24.58%,30.31% at 3 months, which was significantly higher than that in the normal hs-cTnT group. After adjusting for fully confounders, we found a significant association of elevated hs-cTnT level with risks of death or major disability within 3 months after acute ischemic stroke. These results suggested this association was independent of established risk factors, including age, and baseline NIHSS score, and increased serum hs-cTnT elevation could be an independent risk factor of poor outcomes and have prognostic value for death or major disability among patients with acute ischemic stroke.

The evidence on whether increased troponin is associated with stroke was inconsistent: some studies had shown that damage to the right or left insular is associated with higher baseline troponin levels [16, 17], while others had not found any association between insular stroke and troponin levels [18, 19].In our study, 152 were diagnosed as insular stroke, patients with elevated hs-cTnT levels showed significantly higher prevalence of insular stroke, after adjusting for fully confounders, we found a significant association of insular stroke with elevated hs-cTnT level. These results suggested insular damage might contribute to cardiac autonomic dysfunction, the underlying pathophysiological mechanism might be the downregulation of parasympathetic activity, hence the relative up-regulation of sympathetic effects on cardiac function. As a result, this may lead to myocardial injury by contraction zone necrosis or ischemia. In our study showed that the range of hs-cTnT activity was between 14.46~ 58.51 ng/L in patients with increased hs-cTnT levels, which was much lower than that in the myocardial infarction patients. So, hs-cTnT elevation during the acute stage of ischemic stroke, which may be developed by neurally mediated autonomic dysregulation after acute stroke.

Some limitations of this study merit consideration. Firstly, in this study, we relied on a single baseline blood sample and thus we could not account for variations in serum hs-cTnT levels that occur over time, serum hs-cTnT levels should be measured repeatedly to allow longitudinal analysis, which might provide additional information on the development and on its prognostic implications. Secondly, We did not study that association of elevated serum hs-cTnT level with recurrent stroke, which might have an effect on the experimental results. Thirdly, although we adjusted for NIHSS score, which has been show to correlate with infarction volume, we lacked data on infarction volume. Fourth, we lack data on the possible influence of the left and right insular stroke on hs-cTnT and prognosis, respectively, because left and right insular lesion have different influence on the cardiac autonomic function.. In future experiments, we will avoid the aboved limitation, in order to obtain more reliable result.

## Conclusions

Routine serum hs-cTnT measurement in patients with ischemic stroke may provide important novel clinical use. In addition, some studies should be encouraged in regarding to correct cardiac autonomic dysfunction and whether lowering hs-cTnT could prevent the poor outcome of ischemic stroke.

In conclusion, our findings indicated that higher levels of serum hs-cTnT in acute ischemic stroke were associated with increased risk of death or major disability at 3 months. Serum hs-cTnT may have potential predictive value in risk stratification of ischemic stroke.

## Abbreviations

CI: Confidence Interval
hs-cTnT: High sensitivity cardiac troponin
M: Mean
OR: Odds Ratio
SD: Standard Deviation
